# Recurrent central nervous system Rosai-Dorfman disease with KRAS mutation: a case report

**DOI:** 10.1186/s13000-022-01276-7

**Published:** 2023-02-13

**Authors:** Qingyang Wang, Hongxiang Ren, Liyuan Zheng, Juan Wang, Dingrong Zhong

**Affiliations:** 1 Graduate School of Peking Union Medical College, Beijing, 100006 China; 2 Department of Pathology, China-Japan Friendship Hospital, Beijing, 100029 China; 3 Department of Neurosurgery, China-Japan Friendship Hospital, Beijing, 100029 China; 4 Department of Radiology, China-Japan Friendship Hospital, Beijing, 100029 China

**Keywords:** Rosai-Dorfman disease, Sinus histiocytosis with massive lymphadenopathy, KRAS mutation, Central nervous system

## Abstract

**Background:**

Rosai-Dorfman disease (RDD) is a rare, non-Langerhans cell histiocytosis of unknown etiology. we report a very rare case of recurrent central nervous system RDD with KRAS gene mutation and review the literature to improve our understanding of this disease.

**Case presentation:**

A 19-year-old male patient was admitted to our hospital for headache. Cranial magnetic resonance imaging revealed a mass of abnormal signal shadows in the prepontine cistern. The mass was surgically removed and the patient was consequently diagnosed with intracranial Rosai-Dorfman disease. Seven months later, pathological examination confirmed that the RDD had recurred. Next-generation sequencing found KRAS mutation in exon 4 (C.351A > C. P. K117n).

**Conclusion:**

RDD of the CNS has no distinct clinical manifestations and imaging characteristics, and the final diagnosis should be based on the results of the pathological examination. Although RDD is not currently classified as a neoplastic disorder, some evidence of clonality has changed our understanding of it. Follow up examinations over a long period are necessary to determine the efficacy of treatment.

## Background

Rosai-Dorfman disease (RDD), also known as sinus histiocytosis with giant lymphadenopathy, is a rare, non-Langerhans cell histiocytosis of unknown etiology. It usually occurs in the lymph nodes and very rarely in the central nervous system (CNS). RDD is considered a reactive, non-neoplastic disease, and RDD accompanied by genetic mutations is rarely reported in the literature. Here, we report a very rare case of central nervous system RDD with KRAS gene mutation and review the literature to improve our understanding of this disease.

### Case presentation

**Fig. 1:**
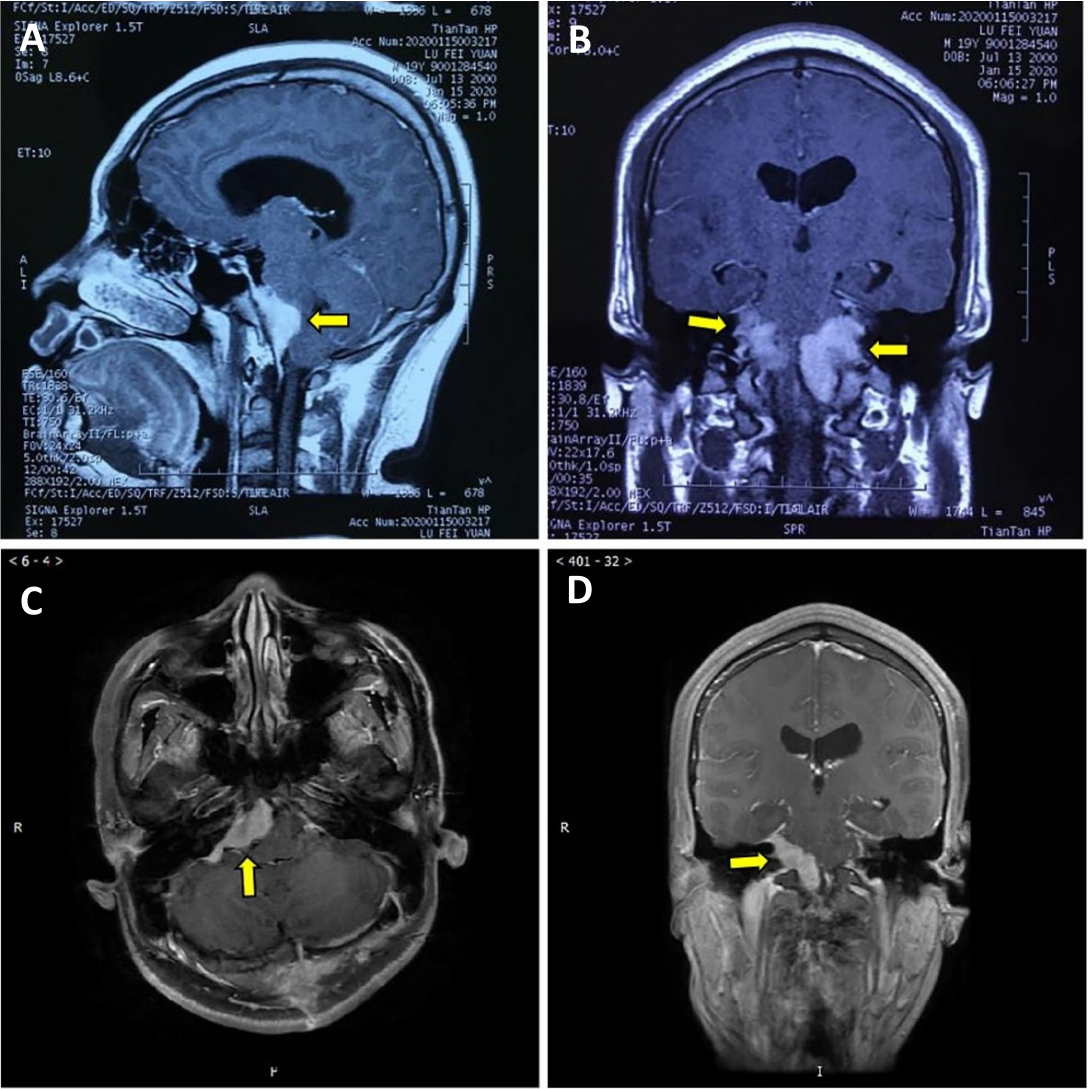
Cranial MRI contrast before and after recurrence. **A** Enhanced MRI (sagittal view) before recurrence; **B** Enhanced MRI (coronal view) before recurrence; **C** Enhanced MRI (transverse sections of the brainstem) after recurrence; **D** Enhanced MRI (coronal view) after recurrence

**Fig. 2:**
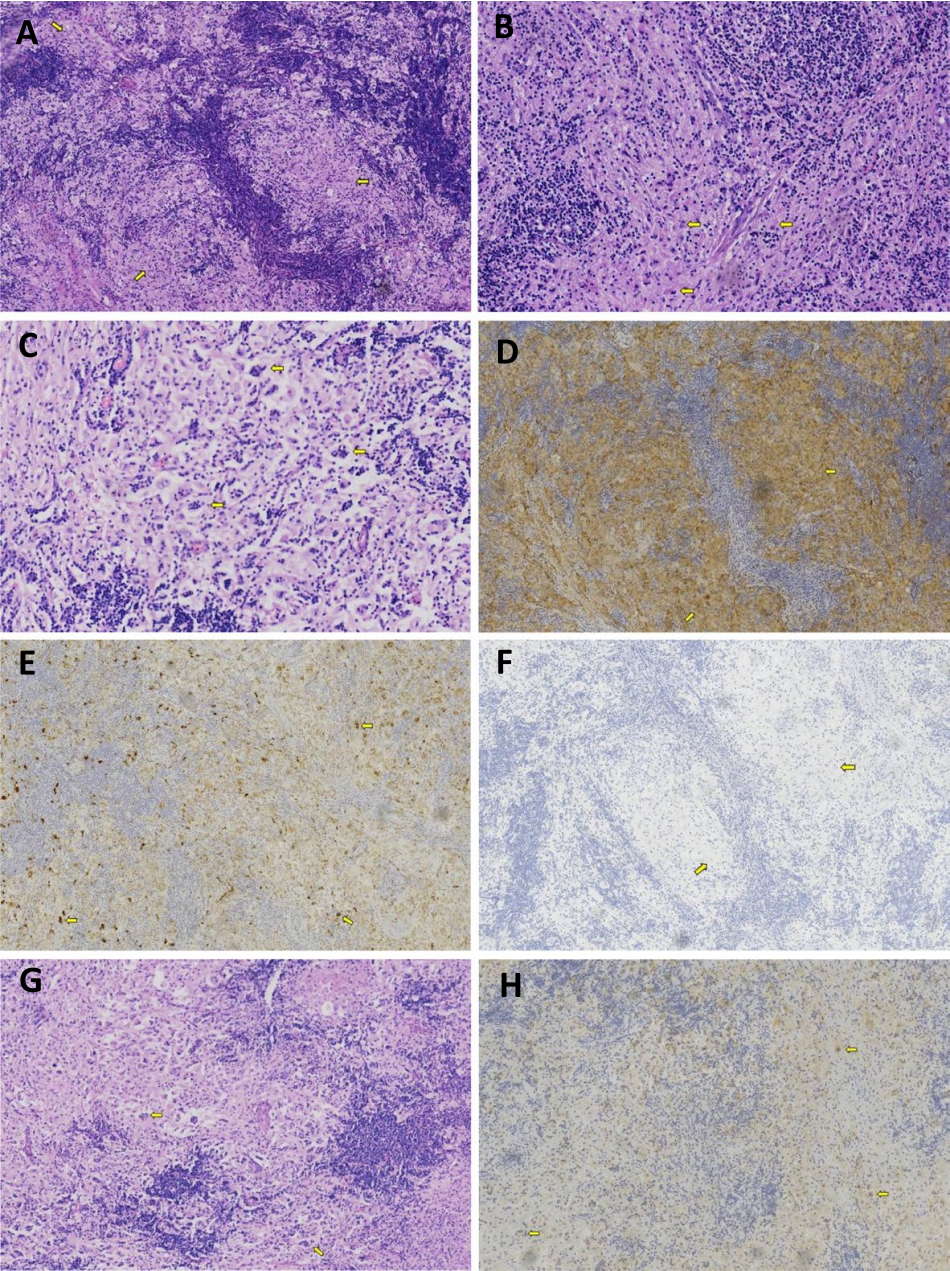
Histopathology and immunohistochemistry. **A** Histiocyte proliferation with lymphocyte and plasma cell infiltration in the background (HE staining, 100X). **B** Histiocytes are rich in cytoplasm, slightly eosinophilic, with round or oval nuclei (HE staining, 400X). **C** Lymphocytes and plasma cells engulfed within histiocytes. **D** Histiocytes are positive for S-100 with immunohistochemical staining. **E** Histiocytes are positive for KP-1 with immunohistochemical staining. **F** Histiocytes are negative for CD1a with immunohistochemical staining. Histopathological findings (**G**) and histiocytes positive for S-100 with immunohistochemical staining (**H**) confirmed the recurrence

A 19-year-old male patient was admitted to the China-Japan Friendship Hospital for headache in January 2020. The patient had developed the headache two months prior to admission, which progressively worsened and was accompanied by tinnitus and dysphagia, but neurological examination revealed no abnormalities. The patient has no family history of cancers. Cranial magnetic resonance imaging (MRI) revealed a mass of abnormal signal shadows in the prepontine cistern. Enhanced MRI showed obvious uniform enhancement, with thickened and strengthened adjacent meninges (Fig. 1). The clinician considered the diagnosis of meningioma and performed partial mass resection. During the operation, a gray-red, hard, and tough mass was found on the ventral side of the brain stem. It had a complete capsule and a clear boundary with the brain tissue. Pathological examination revealed histiocyte proliferation and a large background infiltration of lymphocytes and plasma cells that was also observed in the cytoplasm of some histiocytes. Immunohistochemical staining showed that histiocytes were positive for S− 100, KP-1, and cyclin D1, and negative for CD1a and Langerin, and lymphocytes were positive for CD20 and CD3. Random plasma cells, stained with CD138, were positive for IgG4, and Ki67 staining was 10% positive (Fig. 2). The patient was consequently diagnosed with intracranial Rosai-Dorfman disease. Antibiotics were applied prophylactically to avoid postoperative infection. The patient developed temporary neurological symptoms after surgery, including left facial palsy, hearing loss on the left side, hoarseness and dysphagia, and was given symptomatic supportive treatment. He was followed up regularly in the local hospital and gradually recovered after 2 months.

Seven months later, the patient was readmitted to the hospital because of a headache. Neurological examination revealed left facial palsy, left tongue deviation, right facial hypoesthesia and hypoalgesia, bilateral hearing loss, and ataxia. Cranial MRI showed a mass in the right cerebellopontine foot, extruding the pons and medulla oblongata (Fig. 1). The mass was surgically removed, and pathological examination confirmed that the RDD had recurred (Fig. 2). We used hybridization capture-based next-generation sequencing (NGS) on paraffin-embedded tissues to examine exons, shear regions, and fusion-related introns of 116 genes and found KRAS mutation in exon 4 (C.351A > C. P. K117n).

The patient has received regular follow-up examinations since discharge, and there has been no new recurrence.

## Discussion

Rosai-Dorfman disease is a histiocytic disease that mainly affects the cervical lymph node. Unusual sites, such as retroperitoneal lymph nodes, have also been reported [1]. Approximately 40% of cases may have extranodal tissue or organ involvement, including the upper respiratory tract, gastrointestinal tract, orbit and central nervous system [2]. CNS RDD is rare and only few cases have been reported in literature, with approximately 64.4% occurring in the skull, 22.2% occurring in the spinal canal, and 13.3% affecting both areas simultaneously [3]. It lacks specific clinical manifestations and can cause various neurological symptoms depending on the site of the lesion. There are no obvious imaging characteristics, but the lesion usually presents as an isolated dural mass, similar to meningiomas. Therefore, it is difficult to diagnose RDD based on the clinical manifestations and imaging findings, and it is often misdiagnosed as meningioma, lymphoma, or metastasis before surgery or biopsy.

RDD has unique morphological characteristics and immunohistochemical expression. Morphologically, the expanded sinusoids are filled with cytoplasm-rich and eosinophilic histiocytes. Large infiltrates of lymphocytes and plasma cells are present in the background. Emperipolesis, which means engulfment of lymphocytes, plasma cells, or erythrocytes by the histiocytes, is a feature of RDD. The positive expression of S-100 in the nucleus and cytoplasm of abnormal histiocytes using immunohistochemical staining is an important diagnostic clue.

Differential diagnoses of CNS RDD include: (1) Meningioma that is characterized by the presence of spindle cells with a vortex-shaped arrangement, poor cytoplasm, the lack of emperipolesis, the rare occurrence of lymphoplasmacytic inflammatory cell infiltration in the background, and the observation of psammoma bodies. (2) Langerhans cell histiocytosis (LCH) that is characterized by relatively small tumor cells with nuclear grooves, a lack of emperipolesis, and immunohistochemistry staining that is positive for S-100, CD68, and CD1a. (3) Lymphomas, particularly diffuse large B-cell lymphoma and histiocyte-rich large B-cell lymphoma, are morphologically similar to RDD. The tumor cells express B-cell antigens and clonal rearrangements of immunoglobulin genes can be detected. (4) IgG4-related sclerosing disease (IgG4-RSD) that presents as multiple organ tumor-like lesions with a large infiltration of lymphocytes and plasma cells and an increase in immunohistochemical IgG4-positive plasma cells. (5) Granulomatous disease that is characterized by histiocytes cluster with strong adhesion and less cytoplasm than RDD, and the lack of emperipolesis. Plasma cell aggregation is very rare, except for specific granulomas in diseases such as syphilis. (6) Haemophagocytic lymphohistiocytosis is characterized by uncontrolled activation of cytotoxic T lymphocytes, natural killer cells and macrophages, leading to multi-organ system damage. The diagnosis requires a combination of clinical, histological and biochemical parameters.

RDD is considered a reactive, non-neoplastic disease. In recent years, evidence supporting the clonality of RDD has been found [4–6]. The RAS-RAF-MEK-ERK signaling pathway plays an important role in solid tumors and hematological malignancies. It affects several cellular functions, including proliferation, apoptosis, angiogenesis, migration, and survival [7]. In our case, molecular studies showed KRAS mutation in exon 4 (C.351A > C.P. K117n). KRAS is the most common proto-oncogene, and the RAS protein encoded by KRAS plays a role in signal transduction in intracellular signaling pathways. KRAS mutations occur in a variety of tumors, such as lung, colorectal, and pancreatic cancer [8–10]. KRAS P. K117n is a pathogenic mutation that can cause continuous activation of KRAS protein and the downstream MAPK signaling pathway, thus promoting the occurrence and development of tumors. Garces et al. [6] performed NGS on 21 cases of RDD and one-third of these cases were found to have KRAS and MAP 2 K1 mutations. One of these cases had the same mutation that is reported here. Evidence of the clonality of RDD provides a theoretical basis for the use of targeted drug therapy, and RDD with KRAS mutations responds to MEK inhibitor immunotherapy [6, 11]. Although few cases of recurrent RDD have been reported, it is not clear whether they have genetic mutations. This case is a new addition to the evidence for clonality of RDD.

There have been no cases of CNS RDD self-healing in previously reported cases, and therefore, surgical resection of the lesion is the treatment of choice, particularly in patients with neurological dysfunction related to space-occupying lesions [12]. The lesion in this case has a special growth site and compresses the brain stem and multiple groups of cranial nerves. Therefore, the first operation only partially removed the mass to relieve symptoms and avoid complications of serious neurological damage after surgery. However, the surgery caused some disturbance to the cranial nerves around the mass, resulting in temporary neurological symptoms in the patient. After half a year of follow-up observation, the residual lesion grew again, so a second surgical treatment was performed. Corticosteroids have a limited effect on RDD, but relapse may occur shortly after the interruption of treatment [13]. The efficacy of postoperative radiotherapy and chemotherapy remains unclear [14, 15]. Taking into account the above, the patient was only treated with surgery, without hormone therapy, radiotherapy and chemotherapy.

## Conclusion

In conclusion, we report a rare case of recurrent CNS RDD with KRAS mutation. RDD of the CNS has no distinct clinical manifestations and imaging characteristics, and the final diagnosis should be based on the results of the pathological examination. Although RDD is not currently classified as a neoplastic disorder, some evidence of clonality has changed our understanding of it. Although the prognosis of RDD is good in most cases, there are still rare cases of recurrence. Therefore, follow-up examinations over a long period are necessary to determine the efficacy of treatment.
